# Participation factors for asthma education programs - a cross sectional survey

**DOI:** 10.1186/s12890-019-0979-3

**Published:** 2019-12-19

**Authors:** Oxana Atmann, Klaus Linde, Christoph Werner, Ulrike Dorn, Antonius Schneider

**Affiliations:** Technical University of Munich, TUM School of Medicine, Institute of General Practice and Health Services Research, Orleansstrasse 47, 81667 Munich, Germany

**Keywords:** Asthma patient education, Online asthma education, Health literacy, Electronic health literacy

## Abstract

**Background:**

Although the impact of asthma education on important outcomes (e.g. emergency visits) has been well established, only an estimated quarter of asthma patients in Germany have received patient education yet. The aim of the study was to identify patient factors that could increase participation in asthma education programs.

**Methods:**

This cross-sectional study investigated participation factors and differences between trained (*n* = 64) and untrained (*n* = 65) asthma patients from a large outpatient center in Germany. The survey included answers to asthma-related questions and open questions on patient education as well as such about knowledge of health literacy and eHealth.

**Results:**

Mean age of participants was 55 ± 19 years and 61% were female. Trained patients were more likely to participate in disease management programs (odds ratio (OR) 6.85; 95%CI 2.17–21.59), were more frequently non-smokers (OR 0.07; 95%CI 0.01–0.85) and more often had an asthma action plan (OR 20.2; 1.55–263.41). Open questions’ analysis of untrained asthma patients revealed that patients felt they were not adequately informed about asthma education (37%). About one-third of all patients (27%) showed openness to online asthma education. Analysis of HL and eHealth showed no difference between the groups.

**Conclusion:**

Untrained asthma patients should be informed even more intensively by their physicians about the importance and value of asthma education. Asthma education does not seem to benefit patients’ health literacy. Online asthma education is of interest to approximately one-third of asthma patients. This should be motivation to develop and implement online asthma education concepts.

## Background

The effectiveness of asthma education has been well established in terms of improvement of quality of life, reduction of emergency room visits and health economic costs [1]. Patient education is an intervention that helps chronically ill patients to manage their conditions and the associated burden on their own [2]. According to the German guidelines, each patient with asthma and the indication for a long-term drug therapy should attend an asthma education program. Information about asthma training is available primarily through the patient’s health insurance, general practitioners and pulmonologists. Up to 85% of the costs of currently around 160€ are covered by the patient’s insurance. However, evaluations from routinely collected medical records showed that in Germany only about a quarter of patients participate in asthma education [3]. Recent research identified patient driven barriers to implementing guidelines (e.g. desire for greater empowerment, suboptimal communication between health professionals) and emphasized that patients’ perspectives should be looked at more closely as new guidelines are developed [4]. Currently, most patient education programs are classroom-based and part of the German disease management program (DMP). DMP for asthma showed improvements of symptom frequency, adherence to guidelines, pharmacotherapy, and hospitalization in Germany [3].

The objective of this survey was to identify patient factors that potentially could increase participation in asthma education programs, to understand differences between trained and untrained patients, to gain insights on asthma patients’ perspectives regarding the use of online tools and to assess if health literacy (HL) and electronic health literacy (eHealth literacy) differ between groups.

## Methods

### Study design and participants

The project was designed as a cross-sectional study to examine differences between trained and untrained adult asthma patients regarding asthma education programs. Patients who reported at recruitment that they have participated in asthma education before the study were considered as “trained” patients, whereas patients that have not received asthma education prior the study were defined as “untrained”. The one-site study was conducted at a large outpatient center with 6 pulmonologists, a typical setting for outpatient asthma care in Germany. The study collected data from September through December 2017. To be included, patients had to be 18 years or older, diagnosed with asthma and able to understand German. Exclusion criteria were severe medical conditions. Asthma patients were approached consecutively by a member of the study team (OA) at the reception desk and asked if they wanted to participate. After patients agreed to participate and gave consent to the survey, they were first asked, if they have received asthma training prior to the survey. Depending on their answer, patients received slightly different questionnaires for trained or untrained patients (see below). Afterwards, participants were asked to fill in the questionnaire. Patients filled in the questionnaire without any help or control by the study team member. To achieve similar numbers for both groups, trained patients were also approached after completion of the in-house asthma education program of the center, as their recruitment proved to be more difficult. So far, asthma education in Germany has been offered through evaluated, certified and published in-house programs with predefined content. Patients receive 6 lessons on mainly asthma-self-management through a team of trained physicians and nurses [5].

#### Questionnaire

A questionnaire was developed to understand participation factors for asthma education. Besides using two validated instruments, with questions on health literacy (HLS-EU) and eHealth literacy (eHEALS), demographic and asthma-related questions, questions on motivation to use the internet and open questions were asked. In the open question section trained patients were asked what they liked and did not like about their asthma education. Untrained patients were asked why they did not attend an asthma education program and what they would expect from it. Both groups were asked from where they obtain their information about asthma and if they have openness to attend an online asthma education program. Additionally, the whole sample answered demographic and asthma-related questions (Table 1). Asthma-related questions were: onset of asthma, asthma type and form, asthma medication, if an asthma-action-plan was present, other diseases than asthma, how they obtained information about asthma, when asthma training took place and if they were satisfied (trained group), or if they planned to attend asthma training in the future (untrained). Demographic questions included gender, age, marital status, DMP participation, school diploma and employment. Furthermore, motivation to use digital media and to attend asthma education through different trainers was answered by participants on a 5-point Likert-type scale response: strongly agree, agree, neutral, disagree, strongly disagree.

**Table 1:** Socio-economic factors and asthma-related factors of trained and untrained asthma patients^a^

	Trained (*n* = 64)	Untrained (*n* = 65)	Total sample (*n* = 129)	*p* Value
Age, years (0/0)	55 ± 17	54 ± 21	55 ± 19	0.84
Gender: female (0/0)	37 (58%)	42 (65%)	79 (61%)	0.43
Participation in DMP (11/11)				< 0.001
Yes	25 (47%)	6 (11%)	31 (29%)	
No	15 (28%)	36 (67%)	51 (48%)	
Don’t know	13 (25%)	12 (22%)	25 (23%)	
Marital Status (2/3)				0.08
Married/ with partner	42 (68%)	36 (58%)	78 (61%)	
Single	17 (27%)	15 (24%)	32 (26%)	
Widowed	3 (5%)	11 (18%)	14 (11%)	
School Diploma (2/5)				0.90
Yes	58 (94%)	57 (95%)	115 (94%)	
No	3 (5%)	2 (3%)	5 (4%)	
No answer	1 (2%)	1 (2%)	2 (2%)	
Employment (3/3)				0.50
Yes	34 (56%)	30 (48%)	64 (52%)	
No	25 (41%)	31 (50%)	56 (46%)	
No Answer	2 (3%)	1 (2%)	3 (2%)	
Type of asthma (4/4)				0.31
Allergic	23 (38%)	22 (36%)	45 (37%)	
Non-allergic	17 (28%)	12 (20%)	29 (24%)	
I don’t know	14 (23%)	22 (36%)	36 (30%)	
Other	4 (7%)	5 (8%)	9 (7%)	
Allergic and non-allergic	2 (3%)	0 (0%)	2 (2%)	
Form of asthma (4/3)				0.053
Mild	37 (62%)	39 (63%)	76 (62%)	
Moderate	14 (23%)	21 (34%)	35 (29%)	
Severe	9 (15%)	2 (3%)	11 (9%)	
Smoking (4/2)				0.03
Yes	1 (2%)	11 (18%)	12 (10%)	
Asthma medication (3/1)				0.04
Yes	57 (93%)	52 (81%)	109 (87%)	
Not specified	6 (10%)	4 (6%)	10 (8%)	
Inhaled corticosteroids	34 (56%)	27 (42%)	61 (49%)	
bronchodilator	5 (8%)	7 (11%)	12 (10%)	
Inhaled corticosteroids plus bronchodilator	10 (16%)	10 (16%)	20 (16%)	
I don’t know	1 (2%)	3 (5%)	4 (3%)	
Corticosteroid plus anticholinergics	0 (0%)	1 (2%)	1 (1%)	
corticosteroids plus monoclonal antibodies	1 (2%)	0 (0%)	1 (1%)	
Asthma-action-plan (6/6)				0.001
No	40 (69%)	55 (93%)	95 (81%)	
Yes	13 (22%)	1 (2%)	14 (12%)	
I don’t know	5 (7%)	3 (5%)	8 (7%)	
Other diseases than asthma (9/5)				0.23
None	15 (27%)	21 (35%)	36 (31%)	
One chronic condition	14 (26%)	20 (33%)	34 (30%)	
2 or more chronic conditions	26 (47%)	18 (30%)	44 (38%)	
Not sure	0 (0%)	1 (2%)	1 (1%)	
I inform myself about asthma (more than one answer was possible)				
GP: Yes, (3/2)	18 (30%)	25 (40%)	43 (35%)	0.16
Pulmonary Specialist: Yes (3/3)	56 (92%)	42 (68%)	98 (80%)	0.001
Friends and Family: Yes (3/2)	10 (16%)	7 (11%)	17 (14%)	0.28
Internet	21 (34%)	19 (30%)	40 (32%)	0.38
I was satisfied with my asthma education: Yes	53 (90%)	n.a.	n.a.	
Year of asthma education	2010 ± 9	n.a.	n.a.	
I would like to attend asthma education in the future				
Yes	n.a.	10 (16%)	n.a.	
No	n.a.	17 (27%)	n.a.	
I don’t know	n.a.	37 (58%)	n.a.	

^a^Data are shown with n missing trained/untrained patients, and as mean ± SD or number (%), n.a. = not applicable

The European Health Literacy Survey (HLS-EU) is a measure of subjective Health Literacy (HL) addressing participants’ perceived difficulties in accessing, understanding, appraising and applying information in tasks concerning decision-making in the fields of health care, disease prevention and health promotion. Responses are measured in four categories: fairly difficult, very difficult, fairly easy, very easy [6]. The HLS-EU-Q is a systematically developed and validated instrument [7]. The HL score has a range from 0 to 16, where 0–8 represent inadequate HL, 9–12 problematic HL and 13–16 sufficient HL [8]. The eHealth literacy scale (eHEALS) is an 8-item instrument with a 5-point Likert-type scale response: strongly agree, agree, neutral, disagree, strongly disagree [9]. Items measure patients’ perceived ability to find, evaluate and apply electronic health information to health problems. The authors’ consent to use the instruments was obtained was obtained prior to the study.

#### Analysis

With a sample size of 129 patients the study was powered to detect a standardized mean difference of 0.5 between groups in HLS-EU-Q16 scores with a power of 80% (α = 0.05, two-sided testing). Free text answers to the open questions were grouped into categories of naming similar topics and then were counted by frequency. This was followed by a descriptive evaluation of the quantitative data. To investigate group differences in trained and untrained patients Chi^2^-tests, Mann Whitney U- or Student t-tests were performed. Results were not adjusted for multiple testing. Therefore, the resulting *p*-values must be discussed with caution. To investigate if pre-defined key variables and significantly differing variables between groups were associated independently a multivariate logistic regression analysis was performed. Data are presented as mean ± SD or numbers in percent (%). All analysis was carried out with SPSS 24.0.

## Results

### Sample description

In total, 129 of the 162 approached patients (79%) gave consent, completed and returned the questionnaire. Of those, 64 participants had previously received asthma education (trained patients), while 65 participants had never had asthma education (untrained patients). The mean age was 55 ± 19 years, 61% were female (trained 58%/untrained 65%). Of trained patients 47% were enrolled in DMP, whereas 11% of untrained participants took part in the DMP. Most participants held a school diploma (94%/95%) and half of the participants were employed (56%/48%) (Table 1).

### Asthma-related factors

More than a third indicated an allergic type of asthma (trained 38%/untrained 36%) while more than a third of untrained patients stated not to know their type of asthma (36%) as compared to in the trained group (23%). In terms of degree of asthma the majority reported a mild form of asthma (62%/63%). The proportion of smokers among untrained participants was higher (17%) as compared to trained participants (2%). Of those who did not take asthma medication, the proportion among untrained participants was higher (19%) as compared to trained participants (7%). Only 12% of the whole group had been provided with an asthma action plan and, with the exception of one person, were trained patients. In addition to asthma, one-third of untrained (30%) and almost half of trained patients had two or more other chronic conditions (47%). More trained patients (92%) than untrained ones (68%) sought information about asthma through their pulmonary specialist. Furthermore, one-third of trained patients (30%) received information on asthma from their family physician, as compared to higher numbers among untrained patients (40%), and another third of patients via the internet (34%/30%). In the trained group most were satisfied with their asthma education (90%). Only 16% of those untrained reported that they would attend an asthma education program in the future.

When looking at respondents’ motivation to attend asthma education through different educators, more untrained patients (30%) than trained ones (22%) “strongly agreed” to only attend asthma education when performed by a physician (Table 2).

**Table 2:** Respondents’ motivation to use digital media and to attend asthma education through different trainers^a^

	Trained (*n* = 64)	Untrained (*n* = 65)	Total (*n* = 129)	*p* Value
I only attend asthma education when performed by a physician. (4/11)				
Strongly disagree	19 (32%)	17 (32%)	36 (32%)	0.50
Disagree	10 (17%)	4 (7%)	14 (12%)	
Neutral	7 (12%)	9 (17%)	16 (14%)	
Agree	11 (18%)	8 (15%)	19 (17%)	
Strongly agree	13 (22%)	16 (30%)	29 (25%)	
I am open to participate in asthma education performed by specially trained physician assistants. (5/6)				
Strongly disagree	6 (10%)	11 (19%)	17 (14%)	0.04
Disagree	5 (9%)	4 (7%)	9 (8%)	
Neutral	5 (9%)	12 (20%)	17 (14%)	
Agree	19 (32%)	17 (29%)	36 (31%)	
Strongly agree	24 (41%)	15 (25%)	39 (33%)	
I am open to participate in asthma education performed by specially trained lay patients. (3/7)				
Strongly disagree	9 (15%)	11 (19%)	20 (17%)	0.48
Disagree	17 (28%)	11 (19%)	28 (24%)	
Neutral	9 (15%)	16 (28%)	25 (21%)	
Agree	13 (21%)	14 (24%)	27 (23%)	
Strongly agree	13 (21%)	6 (10%)	19 (16%)	
I would like to do an online asthma education. (6/9)				
Strongly disagree	24 (41%)	19 (34%)	43 (38%)	0.43
Disagree	5 (7%)	5 (9%)	10 (9%)	
Neutral	11 (19%)	13 (23%)	24 (21%)	
Agree	10 (17%)	8 (14%)	18 (16%)	
Strongly agree	8 (14%)	11 (20%)	19 (17%)	
I am motivated to use the internet to learn more about asthma. (3/9)				
Strongly disagree	20 (33%)	15 (27%)	35 (30%)	0.64
Disagree	6 (10%)	5 (9%)	11 (9%)	
Neutral	6 (10%)	8 (14%)	14 (12%)	
Agree	17 (28%)	13 (23%)	30 (26%)	
Strongly Agree	12 (20%)	15 (27%)	27 (23%)	
I would use mobile applications (apps) on my smartphone for asthma. (5/11)				
Strongly disagree	27 (46%)	25 (46%)	52 (46%)	0.006
Disagree	9 (15%)	6 (11%)	15 (13%)	
Neutral	5 (9%)	8 (15%)	13 (12%)	
Agree	15 (25%)	3 (6%)	18 (16%)	
Strongly agree	3 (5%)	12 (22%)	15 (13%)	

^a^Data are shown with n missing trained/untrained patients as mean ± SD or number (%)

Trained patients (41%) are more open than untrained ones (25%) to participate in asthma education when performed by a physician assistant. Fewer untrained patients (10%) than trained patients (21%) were open to participate in asthma education performed through specially trained lay patients. Regarding respondents’ motivation to use digital media, untrained patients (20%) were not only more open than trained ones (14%) to do asthma education offered via the internet (17%), untrained patients (27%) also more often “strongly agreed” to use the internet to learn more about asthma than trained ones (20%). Participants in the untrained group were more open to use mobile applications on their smartphone for asthma (22%) than trained ones (5%).

### Responses to open questions

Untrained patients gave a variety of reasons to not attend asthma patient education. More than a third (37%) reported that they had not been informed about asthma education replying: “I did not know that there is such a thing”. Nearly a fifth of patients (17%) wrote that they did not feel a need to undergo training due to a low burden of disease: “Discomfort is well under my control”. Nine percent stated: “Time constrains”. Untrained patients expected asthma education to increase their asthma-knowledge (28%) saying: “General information about asthma, what to pay attention to and what to do in an emergency event”. Furthermore, they expected to better manage everyday life afterwards (9%), to know “How to deal better with illness and discomfort”, and to gain more information on proper breathing techniques giving responses such as: “New information, everyday tips, and breathing techniques”. Trained patients emphasized a deeper understanding of the disease (34%) after attending asthma education by obtaining more information about asthma saying “All questions were answered in detail” or that they had received “Clarification on what asthma means to me and how it affects me”. Learning both how to take medication on their own (17%), stated as “Asthma-action-plan”, and proper breathing techniques appeared in many responses: “Breathing techniques, proper use of medication, and information on allergies”. Moreover, some trained patients clearly pointed out the positive effect and importance of “Connecting to other people who also have to deal with asthma, [receiving] background information on how to understand the disease” (8%). Only a small number of trained patients answered the evaluation of what they did not like about their patient education, including responses such as “Refresher after three years would be good” (3%). When once again asked about acquiring knowledge about asthma this time in the open question section, both trained and untrained patients answered mainly by their physician (61%/49%), followed by the internet (31%/23%) or print media (16%/11%). The question of whether an Internet-based asthma education program could be an option for them, patients gave heterogeneous answers. Among trained patients (39%) more were not open than open (34%) towards Internet-based asthma education. Among untrained patients an equal part was open to attend (20%) and not open to attend (20%) an online format. Patients open to attend online asthma education typically replied: “Online-based education is interesting to me mainly due to time flexibility” or “Yes, if the costs are covered by the health insurance”. Patients who were not open to asthma education typically replied “Currently I have no need for information; therefore the utility of an online education is questionable” or “I have no Internet”, and “No, I trust my doctor a 100%”. Some patients open towards online education mentioned the conditions under which they would attend one: “Yes, if compatible with [my] everyday life” (Table 3).

**Table 3:** Responses to open questions – main categories^a^

Variable	Response
Reasons to not attend asthma education (Untrained, 65)	• No information obtained (24, 37%) • Low feeling of discomfort (11, 17%) • No time (6, 9%)
Expectations of patient education (Untrained, 65)	• More asthma knowledge (18, 28%) • Better management of everyday life (6, 9%) • No expectations (6, 9%)
Positive characteristics of patient education (Trained, 64)	• Information about asthma (22, 34%) • To learn the handling of cortisol (10, 17%) • Dealing with chronical condition (8, 13%) • Connecting to other affected patients (5, 8%)
Negative characteristics of patient education (Trained, 64)	• None (2, 3%) • Refreshers necessary (2, 8%) • Too long (1, 2%)
Obtaining information about asthma (Trained, 64)	• Physician (39, 61%) • Internet (20, 31%) • Print Media (10, 16%)
Obtaining information about asthma (Untrained, 65)	• Physician (32, 49%) • Internet (15, 23%) • Print Media (7, 11%)
Openness to attend online education and condition for online asthma education (Trained, 64)	• Open to attend (22, 34%) • Not open to attend (25, 39%) • Compatibility with everyday life (1, 2%) • Don’t know (1, 2%)
Openness to attend online education and condition for online asthma education (Untrained, 65)	• Open to attend (13, 20%) • Not open to attend (13, 20%) • Compatibility with everyday life (6, 9%) • Don’t know (6, 9%)

^a^Data are shown as total number of patients in both groups mentioning the category, number (%), more than one answer was possible

### Health literacy and eHealth literacy

HL assessed with the HLS-EU-Q16 showed a sum score of 13 ± 3, demonstrating a HL between “problematic” and “sufficient”. EHealth literacy administered with the eHEALS showed a middle range with a score of 3 ± 1. No group differences could be shown for HL and eHealth literacy in the respective sum score (Table 4).

**Table 4:** Sum score HLS-EU-Q16 and eHEALS^a^

Sum score	Trained	Untrained	Total Sample	*p* Value
HLS-EU-Q16 score	13 ± 3	13 ± 3	13 ± 3	0.16
eHEALS all Items	3 ± 1	3 ± 1	3 ± 1	0.74

*HLS-EU-Q16* European Health Literacy Survey, *eHEALS* electronic health literacy scale

^a^Data are shown as mean ± SD

### Regression analysis

Multivariate regression analysis showed three variables that were more likely for trained patients than untrained ones. Trained patients were more likely to participate in disease management programs (odds ratio (OR) 6.85; 95% CI 2.17; 21.59), were more frequently non-smokers (OR 0.07; 95% CI 0.01–0.85) and more often had an asthma action plan (OR 20.2; 95% CI 1.55; 263.41). No significance associated with group status was shown regarding age, gender, health literacy, perceived asthma control and interest in an internet-based training program (Table 5).

**Table 5:** Associated factors of asthma education in multivariate regression analysis (*n* = 108). *R*^2^ = 0.41 (Nagelkerke)

Variable	OR (95%CI)	*p*-Value
Age (per year)	1.00 (0.98–1.03)	0.78
Gender (female)	0.92 (0.34–2.49)	0.87
DMP utilization (yes)	6.85 (2.17–21.59)	0.001
Smoking (no)	0.07 (0.01–0.85)	0.04
Asthma action plan	20.2 (1.55–263.41)	0.02
Health Literacy (HLS-EU-Q16)	1.01 (0.88–1.17)	0.86
Asthma subjectively under control	1.40 (0.92–2.13)	0.12
Interest in internet-based asthma education	2.62 (0.90–7.62)	0.08

*HLS-EU-Q16* European Health Literacy Survey, *eHEALS* electronic health literacy scale, *OR* Odds Ratio

## Discussion

Key findings suggest that untrained asthma patients were not sufficiently informed about asthma education programs (37%), did not attend DMP (67%), did not have an asthma action plan (93%), and were more often smokers (18%) as compared to trained ones (2%). Asthma patients continue to see their physicians as the most important source of information on asthma.

It is noteworthy, that only a small number of participants had an asthma action plan (trained 22%/untrained 2%). Low numbers of patients with an asthma action plan are also found in previously published research [10]. As many studies show why the asthma action plan is important for outcomes, reasons for the small percentage of patients could be a lack of healthcare professionals to implement guidelines in a busy practice, lack of appropriate materials or unclear roles in terms of self-management [11]. Additional reasons for this low number are shown in a qualitative survey by Cabana et al. It revealed that at times neither patients nor doctors were enthusiastic about self-management plans and sometimes even ambivalent about their usefulness and relevance [12]. Moreover, even if patients were motivated to use self-management-plans, Jones et al. reported little sustained use and/or the believe that the plans were largely irrelevant [13]. Attitudes associated with these views could reflect the gap between the physicians’ concept of the “responsible asthma patient” and the patient’s personal view [13]. Another underlying factor might also be paternalistic approaches in the health care system and the expectations of physicians that patients follow their ideas and norms [14]. One being the importance of asthma education as viewed by health professionals that can contradict patients’ own ideas about how to deal with their chronic disease [15]. As the self-management-plan should regularly be part of asthma management, especially in DMP and trained asthma patients, reasons for these low numbers should be examined in further research. In our study, the majority of participants did not smoke. However, the proportion of smokers among untrained patient was higher than among trained ones. This could be due to selection bias, as trained patients seemed to be more affected by asthma. Therefore, those patients possibly more often take part in training programs and maybe are more likely to quit smoking. On the other hand, this association could also suggest that asthma patients who smoke are aware or perceive that tobacco smoke is a factor that may trigger or worsen their asthma symptoms. Thus, such patients might consider it useless to them to attend an asthma education program if they have not previously quit smoking. Care should be exercised when interpreting these results as the questionnaire did not focus on smoking behavior and number of patients was limited. Nonetheless, our findings may indicate that asthma education could increase awareness of smoking cessation.

It is worthy of mentioning, that only 16% of untrained patients plan to attend asthma education in the future. This might be an expression of motivational lack due to mild symptoms, lack of practice’s organization or lack of information on the part of the health professional or health organization [16]. The majority of untrained asthma patients (58%) stated that were unsure if they would attend asthma education in the future. With tailored action by health care professionals and organizations in the health sector (e.g. health insurance companies), there is a possibility to motivate a part of these still “undecided” patients to attend asthma education. This group should be targeted especially to increase asthma education rates among asthma patients. Furthermore, when looking to increase rates, organizational variables in the background should be considered as well, taking into account lack of time, resources and insufficient training of health professional regarding patient education, as well as necessary improvements of IT-systems to support the physician-patient relationship [17].

A regular check of patients’ asthma self-management by health professionals is required in the German guidelines. In Germany, this is mainly performed by primary care physicians; in other countries, this role is often successfully taken by specialized nursing staff or even specially trained lay educators [18, 19]. In this survey, one-third was open to attend asthma education performed by physician assistants (33%), and only less than a fifth open to attend asthma education performed by specially trained lay patients (16%). Should the implementation of recommended guidelines continue to be less optimal, alternative self-management support by health care professionals should as well be explored, e.g. through lay educators, digital media.

Often, asthma is accompanied by allergic diseases (e.g. allergic rhinitis) and other respiratory disorders (e.g. sleep apnea), as well as by metabolic, cardiovascular and mental illnesses [20, 21]. In the present survey, one-third reported having one more chronic disease (trained 26%/ untrained 33%). Almost half of the trained patients reported two or more chronic diseases (47%), whereas only a third of untrained patients reported the same (30%). In most cases, patient education programs are conducted specifically focused on one chronic disease and rarely or not all take into account the presence of multiple chronic diseases [21]. However, patients with three or more chronic conditions are 14 times more likely to be hospitalized than people without a chronic condition. People with comorbidities spend 25 times more nights in the hospital than adults without a chronic condition [22]. As studies show that chronic diseases have many similarities, chronic education programs targeting various chronic disease should be broader implemented [23]. It could be demonstrate that these educations are superior to the usual healthcare delivery and result in fewer hospital days [24, 25].

Findings of this study showed no group difference regarding health literacy and eHealth literacy. To date neither health literacy nor eHealth literacy are integrated in usual asthma education programs [26]. Since health literacy should be the basis for successful health care navigation, it should be an integral part of any patient education for chronic patients. In addition, health care professionals themselves seem to lack of awareness and practice of health literacy and eHealth [27]. To date there is no consensus on how health literacy practices should be implemented in the education of health professionals [28]. Additionally, ideal strategies for health professionals to communicate health literacy practices to their patients remain unclear [29].

The trend towards the widespread use of digital media is hardly considered in asthma education in Germany [26]. Furthermore, dealing with digital media can be a challenge for both patients and health care providers [30]. The results of this study suggest that about one-fifth was motivated to use the internet to learn more about asthma, whereas respondents’ motivation to utilize online asthma education was quiet low (17%). This may be due to participants’ mean age of 54 years suggesting low motivation to use the internet or even general low interest in asthma education. In addition, motivation to use a mobile application for asthma was even lower (13%). Nevertheless, one-third of the entire group was open to online asthma education. Recent surveys show that the digital divide in Germany is decreasing in terms of age, socioeconomic status, gender, education and rural versus urban populations. Currently, 84% of the German population are online. The number of over 60 years and older people increased from 4% in 2001 to 45% in 2018 [31].

Limitations of this exploratory, cross-sectional survey have to be taken into account when interpreting the data. Generalizability of our findings might be compromised by the specific characteristics of the German healthcare system, e.g. easy access to specialists without gate-keeping by general practitioners, and the fact that patients were recruited at a single center. While our study was powered to detect moderately large differences between groups for ordinal and continuous variables, the number of patients was relatively small. Therefore, existing differences, particularly in nominal variables, might have been missed. On the other hand, the statistically significant differences between the groups found in univariate analyses must be interpreted with caution, as we did not adjust for multiple testing. This limitation, however, does not apply to the findings of the multivariate regression analysis. A strength of our study is the additional collection of answers to open questions among all participants.

Although this survey does not allow statements on the causality of attendance of asthma education programs, it does provide much needed descriptive data to understand asthma patients’ experience on education programs. Useful conclusions can be drawn to understand asthma patient’s experience of asthma education programs.

## Conclusion

Health care professionals should address the importance of asthma action plans and asthma education programs even more proactively. More research should be done on possibly conducive factors for sustainable asthma education programs, and how to better address the issue of health literacy and eHealth in asthma education programs.

## Data Availability

Only aggregated and anonymized data are available for members of the research group or journals based on agreement with patients and our ethics committee. It is not possible to share any independent patient level data.

## Abbreviations

DMP: Disease management program
eHealth: Electronic health literacy
GP: General Practitioner
HL: Health literacy
HLS-EU: European Health Literacy Survey
OR: Odds Ratio
